# Supplementary figures and images for: Generational mutation patterns in a honey bee Deformed wing virus via infectious clones
Source: PLoS One. 2025 Nov 19;20(11):e0337191. doi: 10.1371/journal.pone.0337191 (PMC12629483; doi:10.1371/journal.pone.0337191)

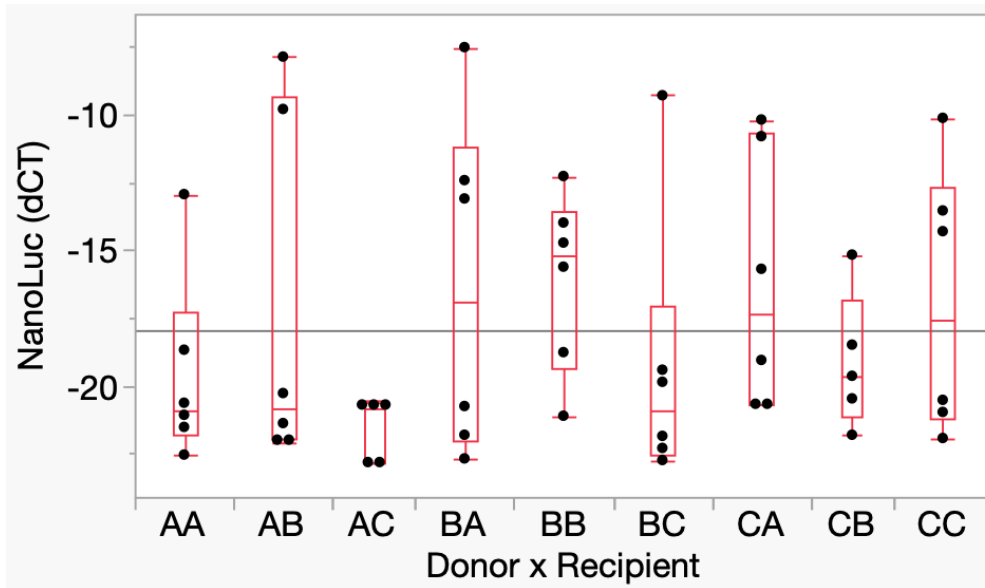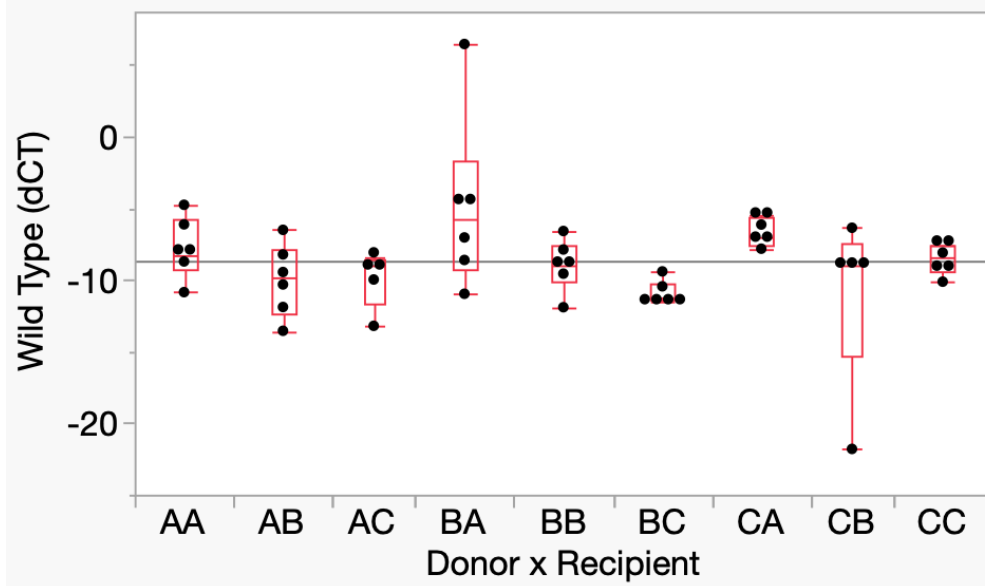

Supplement: S1 Fig — (PDF) [file pone.0337191.s001.pdf]

Amplicon Sequence Variants (mean)

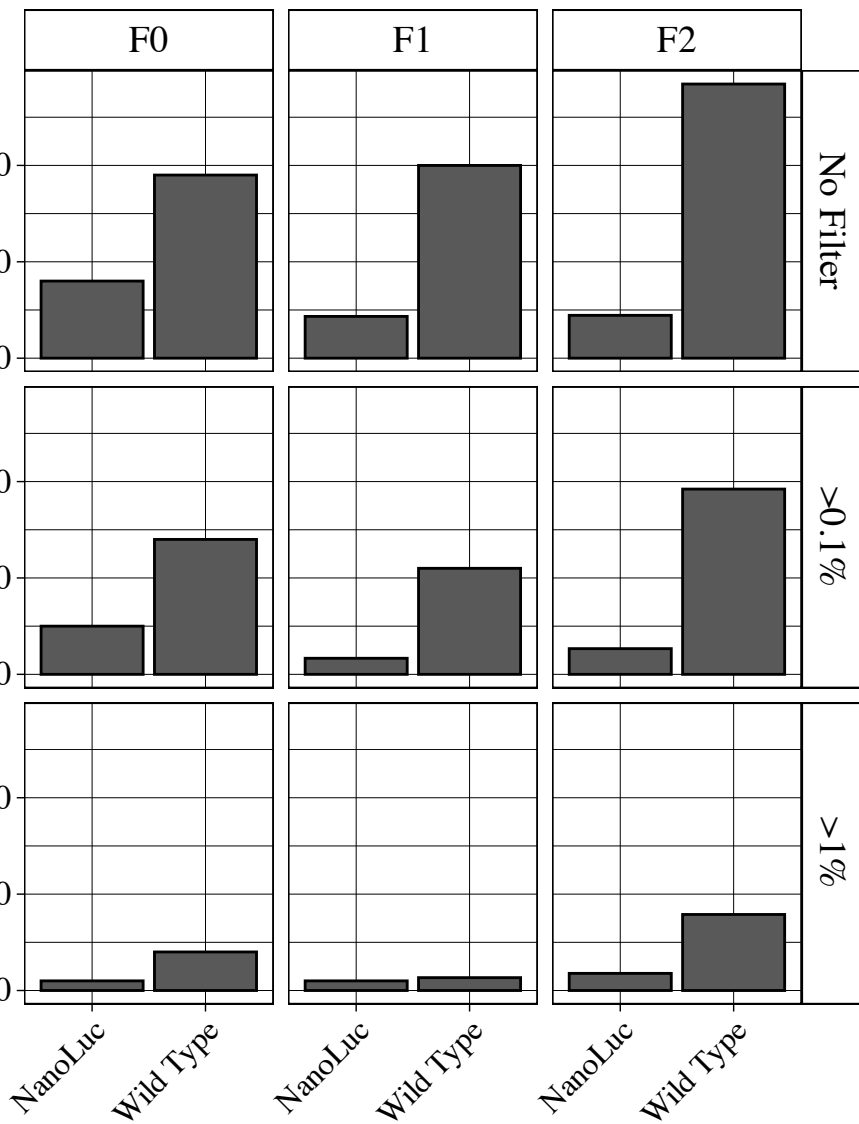

Supplement: S2 Fig — (PDF) [file pone.0337191.s002.pdf]

ML tree with ASVs > 1%

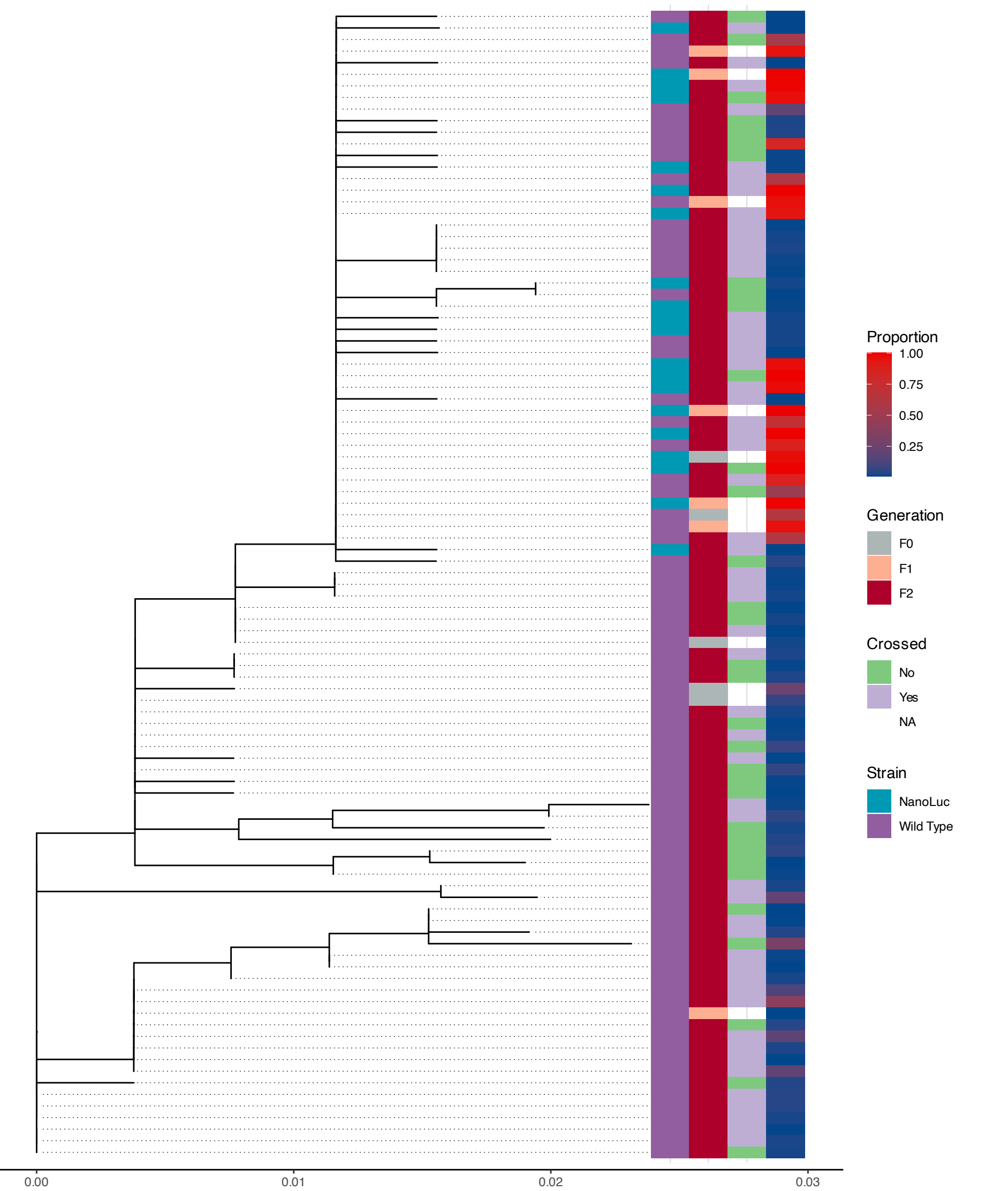

Supplement: S3 Fig — Legend further identifies ASVs by their strain, injection generation, cross fostering status, and precise proportional representation. (PDF) [file pone.0337191.s003.pdf]

ML tree with ASVs > 0.1%

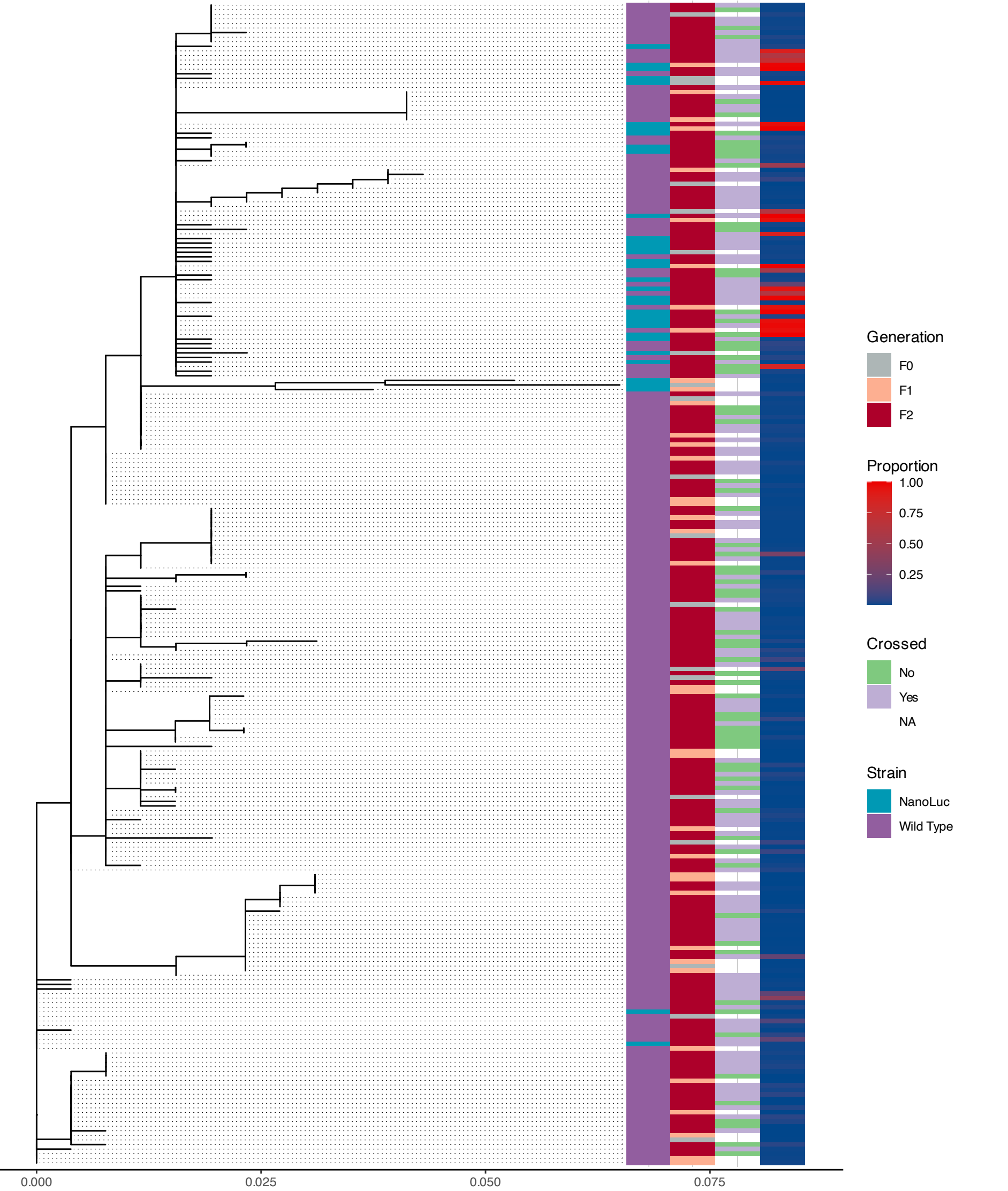

Supplement: S4 Fig — Legend further identifies ASVs by their strain, injection generation, cross fostering status, and precise proportional representation. (PDF) [file pone.0337191.s004.pdf]

ML tree with all ASVs

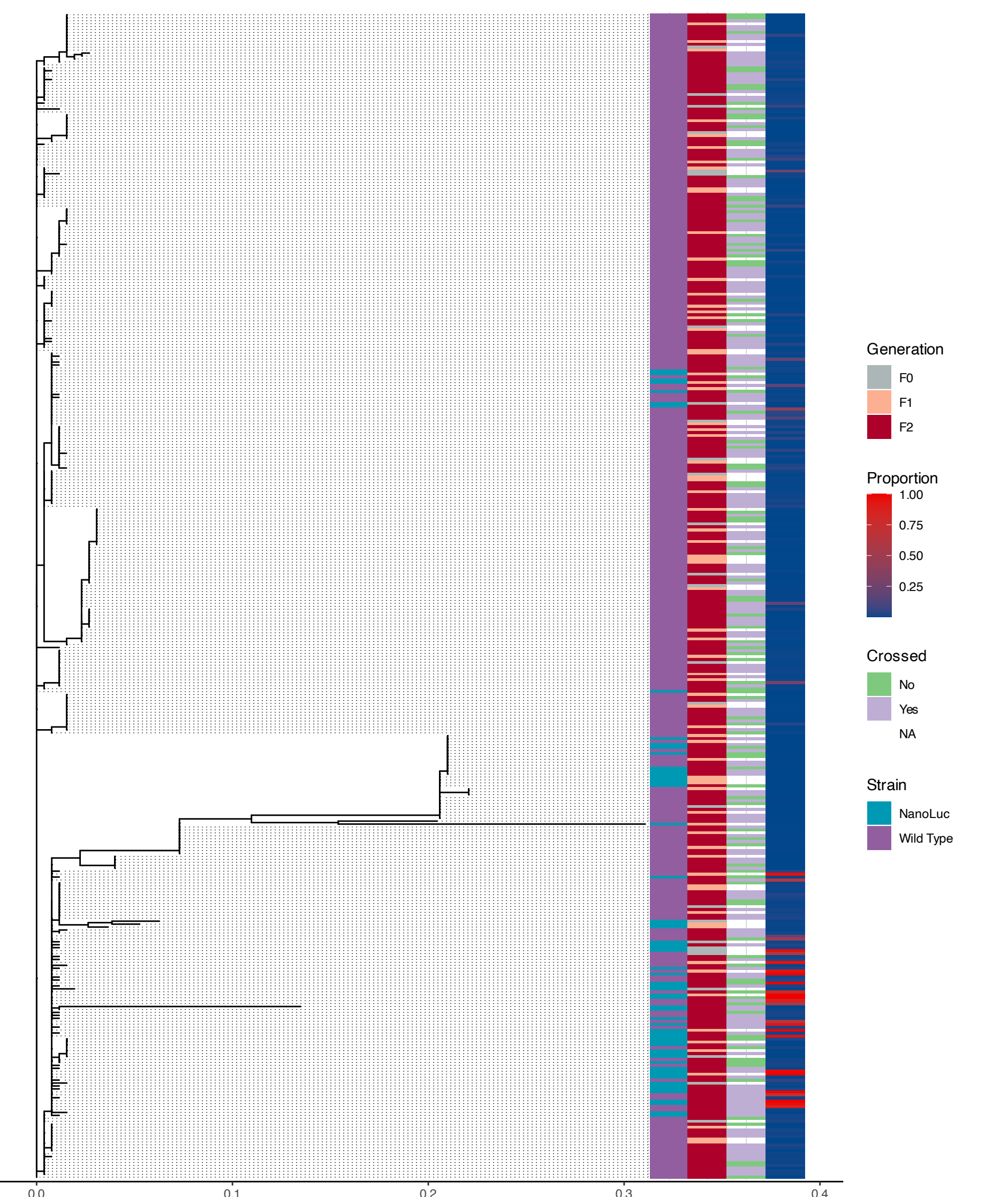

Supplement: S5 Fig — Legend further identifies ASVs by their strain, injection generation, cross fostering status, and precise proportional representation. (PDF) [file pone.0337191.s005.pdf]
