# Supplementary material for: Generational mutation patterns in a honey bee Deformed wing virus via infectious clones
Source: PLoS One. 2025 Nov 19;20(11):e0337191. doi: 10.1371/journal.pone.0337191 (PMC12629483; doi:10.1371/journal.pone.0337191)
Supplement: S2 Table — Counts of unique amplicon sequence variants are broken down into three categories of proportional representation: > 1%, > 0.1%, and no filter (total). (PDF) [file pone.0337191.s007.pdf]

Strain	Generation	Sample ID	Source Colony	End Colony	Cross Fostered	Library Size	Amplicon Sequence Variants		
							> 1%	> 0.1%	Total
NanoLuc	F0	P1 S14				99964	1	5	8
	F1	P1 S1	A			83281	1	2	5
		P1 S2	B			150251	1	2	6
		P1 S3	C			41234	1	1	2
	F2	P1 S4	A	A	No	77440	3	4	5
		P1 S5	A	B	Yes	64092	2	2	5
		P1 S6	A	C	Yes	141635	3	7	13
		P1 S7	B	A	Yes	11507	1	1	2
		P1 S8	B	B	No	35420	1	2	5
		P1 S9	B	C	Yes	1755	1	2	2
		P1 S10	C	A	Yes	58301	3	4	5
		P1 S11	C	B	Yes	642	1	1	1
		P1 S12	C	C	No	5153	1	1	2
Wild Type	F0	P3 S14				21235	4	14	19
	F1	P3 S1	A			27572	1	11	19
		P3 S2	B			92896	1	11	23
		P3 S3	C			23256	2	11	18
	F2	P3 S4	A	A	No	67983	8	21	32
		P3 S5	A	B	Yes	85023	10	22	29
		P3 S6	A	C	Yes	120524	4	14	28
		P3 S7	B	A	Yes	40669	9	24	32
		P3 S8	B	B	No	29486	5	15	21
		P3 S9	B	C	Yes	161368	8	19	33
		P3 S10	C	A	Yes	107602	6	16	29
		P3 S11	C	B	Yes	15219	6	14	18
		P3 S12	C	C	No	27734	15	28	34
