# Supplementary material for: Generational mutation patterns in a honey bee Deformed wing virus via infectious clones
Source: PLoS One. 2025 Nov 19;20(11):e0337191. doi: 10.1371/journal.pone.0337191 (PMC12629483; doi:10.1371/journal.pone.0337191)
Supplement: S1 File — (DOCX) [file pone.0337191.s008.docx]

P1 Forward - NanoLuc

CCTGCTGTTCCGAGTAACCA

P1 Reverse - NanoLuc

TCGAGATGTGATTGTGGCGT

P3 Forward - Wild Type

CCTAATCCAGGACCTGATGGC

P3 Reverse - Wild Type

CTTCCATATCGCCTCGCCAA
